# Specific inhibition of *Streptococcus bovis* by endolysin LyJH307 supplementation shifts the rumen microbiota and metabolic pathways related to carbohydrate metabolism

**DOI:** 10.1186/s40104-021-00614-x

**Published:** 2021-08-04

**Authors:** Hanbeen Kim, Tansol Park, Inhyuk Kwon, Jakyeom Seo

**Affiliations:** 1 Department of Animal Science, Life and Industry Convergence Research Institute, Pusan National University, 1268-50 Samrangjin-ro, Miryang, 50463 Republic of Korea; 2 Department of Animal Science and Technology, Chung-Ang University, Anseong-si, Gyeonggi-do 17546 Republic of Korea; 3 EASY BIO, Inc., Seoul, 06253 South Korea

**Keywords:** Endolysin, Rumen microbiota, Ruminal acidosis, *Streptococcus bovis*

## Abstract

**Background:**

Endolysins, the bacteriophage-originated peptidoglycan hydrolases, are a promising replacement for antibiotics due to immediate lytic activity and no antibiotic resistance. The objectives of this study were to investigate the lytic activity of endolysin LyJH307 against *S. bovis* and to explore changes in rumen fermentation and microbiota in an *in vitro* system. Two treatments were used: 1) control, corn grain without LyJH307; and 2) LyJH307, corn grain with LyJH307 (4 U/mL). An* in vitro* fermentation experiment was performed using mixture of rumen fluid collected from two cannulated Holstein steers (450 ± 30 kg) and artificial saliva buffer mixed as 1:3 ratio for 12 h incubation time. *In vitro* dry matter digestibility, pH, volatile fatty acids, and lactate concentration were estimated at 12 h, and the gas production was measured at 6, 9, and 12 h. The rumen bacterial community was analyzed using 16S rRNA amplicon sequencing.

**Results:**

LyJH307 supplementation at 6 h incubation markedly decreased the absolute abundance of *S. bovis* (approximately 70% compared to control, *P* = 0.0289) and increased ruminal pH (*P* = 0.0335) at the 12 h incubation. The acetate proportion (*P* = 0.0362) was significantly increased after LyJH307 addition, whereas propionate (*P* = 0.0379) was decreased. LyJH307 supplementation increased D-lactate (*P* = 0.0340) without any change in L-lactate concentration (*P* > 0.10). There were no significant differences in Shannon’s index, Simpson’s index, Chao1 estimates, and evenness (*P* > 0.10). Based on Bray-Curtis dissimilarity matrices, the LyJH307 affected the overall shift in microbiota (*P* = 0.097). LyJH307 supplementation induced an increase of 11 genera containing *Lachnoclostridium*, WCHB1–41, unclassified genus Selenomonadaceae, *Paraprevotella*, vadinBE97, *Ruminococcus gauvreauii* group, *Lactobacillus*, *Anaerorhabdus furcosa* group, *Victivallaceae*, *Desulfuromonadaceae*, and *Sediminispirochaeta*. The predicted functional features represented by the Kyoto Encyclopedia of Genes and Genomes pathways were changed by LyJH307 toward a decrease of carbohydrate metabolism.

**Conclusions:**

LyJH307 caused a reduction of *S. bovis* and an increase of pH with shifts in minor microbiota and its metabolic pathways related to carbohydrate metabolism. This study provides the first insight into the availability of endolysin as a specific modulator for rumen and shows the possibility of endolysin degradation by rumen microbiota.

**Supplementary Information:**

The online version contains supplementary material available at 10.1186/s40104-021-00614-x.

## Background

The use of high amounts of non-fiber carbohydrate (NFC) is generally accepted by farmers to meet the nutritional energy requirements of high-performance cattle; however, this strategy often carries nutritional disorders, including the occurrence of ruminal acidosis. Generally, ruminal acidosis can be divided into two types, subacute ruminal acidosis (SARA) and acute acidosis [1]. The outbreak of ruminal acidosis starts from increased inflow of NFC into the rumen, and it can stimulate the overgrowth of amylolytic bacteria, which induce the increase of volatile fatty acids (VFA) and lactate production and decrease ruminal pH [2]. In this condition, excessive production of VFA with a concomitant increase of lactate fermenting bacteria produces SARA; however, improper growth of lactate fermenting bacteria can induce acute acidosis by the accumulation of lactate in the rumen [1, 3]. Both types of acidosis can cause a decrease in production performances (average daily gain, growth rate, and milk yield) and create an abnormal balance of rumen microbiota, causing significant economic damage to farmers [4].

*Streptococcus bovis* is one of the main ruminal amylolytic bacteria that can produce lactate as the major fermentation product under low ruminal pH conditions. Under a high grain diet, *S. bovis* can generate more ATP per hour than other ruminal amylolytic bacteria, and *S. bovis* features not only high acid resistance, but also rapid growth rate [5, 6]. Therefore, *S. bovis* is one of the critical initiators of ruminal acidosis.

Great efforts have been undertaken to prevent an outbreak of ruminal acidosis, such as a change of feeding strategies (e.g.*,* a gradual increase of concentration source) [7], use of buffering agent [8], or feeding rumen modulators such as chemical agent (α-amylase and glucosidase inhibitor) [9]. In addition, there have been a few studies attempting to inhibit *S. bovis* specifically by immunization [10, 11] and bacteriocin [12]. However, to the best of our knowledge, there have been no studies attempting to modulate ruminal acidosis by endolysin.

Endolysin is bacteriophage-originated peptidoglycan (PG) hydrolases that can break down the PG layer of the host from within the bacteria during the final stage of the replication cycle [13]. In general, endolysin targeting gram-positive bacteria comprises at least two conserved functional domains, N-terminal enzymatically active domain (EAD) and C-terminal cell wall binding domain (CBD). The CBD affects host specificity by recognizing and binding to the host PG layer, and the EAD can degrade the PG layer when increased endolysin-PG proximity is made by CBD [14]. The number of studies related to endolysin development has been improved by the emergence of multi-drug resistant bacteria, which render many types of antibiotics ineffective. Many researchers have shown that endolysin can lyse gram-positive bacteria when endolysin is administered externally because of the absence of an outer membrane such as a lipopolysaccharide layer [15–17]. Therefore, endolysin is a promising replacement for antibiotics. In the food and biomedical health fields, many endolysin applications have tried to solve the problem of antibiotic resistance [18]; however, in the livestock science field, to the best of our knowledge, limited trials have been reported on endolysin development for treating mastitis-associated bacteria, specifically *Staphylococcus aureus* [19], and infectious bacteria on fresh cheese, specifically *Listeria monocytogenes* [20]. Moreover, there have been no attempts to apply endolysin for modulating ruminal metabolic disease via the specific control of ruminal bacteria in the rumen.

In a previous study, we developed endolysin LyJH307 with high lytic activity against *S. bovis* [15]. Endolysin LyJH307 has a distinct combination of conserved domain consisting of N-terminal NlpC/P60 superfamily (EAD) and C-terminal Zoocin A target recognition domain superfamily (CBD), and the endolysin LyJH307 had potent lytic activity in a variety of condition (pH, 5.0–6.0; temperature, 25–55 °C; NaCl concentration, 0–250 mmol/L). Moreover, it confirmed that endolysin LyJH307 retained lytic activity against the *S. bovis* in single culture media. Therefore, we expected that endolysin LyJH307 would have potent lytic activity against the *S. bovis* group in rumen condition.

Therefore, the objectives of the present study were to (1) investigate the lytic activity of endolysin LyJH307 against *S. bovis* in an *in vitro* system, and (2) explore the changes in rumen fermentation and microbiota. This study hypothesized that the endolysin LyJH307 might induce the reduction of *S. bovis* in an *in vitro* system, thereby increasing ruminal pH.

## Methods

The protocols for this study concerning animal use were reviewed and approved by the Animal Research Ethics Committee of Pusan National University (Pusan, Korea, PNU-2020-2827).

### Preparation of recombinant endolysin LyJH307

Recombinant LyJH307 was cloned in *Escherichia coli* BL21 (DE3), and the effect of lytic activity on the *S. bovis* group was tested following the methodology described by Kim, Lee, Kwon (15). To produce the recombinant endolysin LyJH307, the transformant was grown in Luria-Bertani medium (Difco Laboratories Inc.) until the optical density at 600 nm (OD_600nm_) reached 0.4. After that, isopropyl-β-D-thiogalactoside (1 mmol/L) was added to the medium, and the cells were further incubated for 4 h at 37 °C. Harvested cells were suspended in lysis buffer (50 mmol/L NaH_2_PO_4_, 300 mmol/L NaCl, and 10 mmol/L imidazole at pH 8.0), and lysed by sonication (KYY-80, Korea Process Technology Co., Ltd., Seoul, Korea). After centrifugation at 10,000×*g* for 15 min, the supernatant was passed through Ni-NTA Agarose (Qiagen GmbH, Hilden, Germany) and the recombinant LyJH307 was purified as described by the manufacturer, and resolved by sodium dodecyl sulfate-polyacrylamide gel electrophoresis. The purified endolysin was incubated with 5 mmol/L ethylenediaminetetraacetic acid at 25 °C for 30 min to chelate divalent cations attached to the endolysin. The ethylenediaminetetraacetic acid-treated LyJH307 was concentrated with an Amicon Ultra-4 (10 kDa, Merck KGaA, Darmstadt, Germany), and the purified LyJH307 was mixed with elution buffer (50 mmol/L NaH_2_PO_4_, 300 mmol/L NaCl, and 10 mmol/L CaCl_2_ at pH 8.0).

### Experimental diets and chemical analysis

To induce the acidosis condition within 12 h under *in vitro* fermentation, we selected corn grain as the experimental substrate. The chemical composition of the corn was as follows: dry matter (DM), 88.5%; crude protein (CP), 8.4%DM; neutral detergent fiber (aNDF), 12.4%DM; acid detergent fiber (ADF), 5.3%DM; ether extract (EE), 4.7%DM; starch, 70.6%DM; sugar, 3.4%DM; non-fibrous carbohydrate, 74.0%DM; ash, 1.1%DM; and lignin, 1.7%DM. Before chemical analysis was conducted, the corn grain was dried at 60 °C for 96 h and ground with a cyclone mill (Foss Tecator Cyclotec 1093, Foss, Hillerød, Denmark) fitted with a 1 mm screen. The DM (#934.01), CP (#990.03), ADF (#973.18), and ash (#942.05) were analyzed by AOAC international methods [21]. The EE (#2003.05) was assessed using AOAC international methods [22]. The CP was calculated by multiplying the nitrogen content by 6.25, and total nitrogen was measured by the Kjeldahl method with a nitrogen combustion analyzer (Leco FP-528 Leco, MI, USA). The aNDF and lignin were analyzed [23] to determine the fiber content. Heat-stable amylase (α-amylase) was used to estimate aNDF and was expressed inclusive of residual ash. The NFC of the experimental diet was estimated as follows: NFC = [100 – ash – EE – CP – aNDF].

### Experimental treatment

A complete randomized design was used for the experiment, with treatment as the main effect. Two experimental treatments were used as follows: (1) Control diet (CON) containing corn grain with elution buffer, and (2) endolysin LyJH307 treatment (LyJH307) containing corn grain with endolysin LyJH307 (4 U/mL). The volume of elution buffer supplemented was the same as that of the endolysin in the LyJH307 treatment.

### *In vitro* rumen fermentation

*In vitro* fermentation was undertaken using the rumen fluid (1 L per animal) collected from two cannulated Holstein steers (body weight = 450 ± 30 kg) before the morning feed at the Center for Agriculture Research, Pusan National University, Korea. Animals were fed a diet of 600 g/kg Timothy hay and 400 g/kg of a commercial concentrate mix. The rumen fluid was collected before the morning feeding time, mixed, transferred into a thermos bottle, and immediately transported to the laboratory. The rumen contents were filtered through four layers of cheesecloth and mixed with 3× volumes of in vitro rumen buffer solution [24]. Approximately 1 g of the ground experimental substrate was placed into pre-weighed nylon bags (R510, Ankom Technology, NY, USA). All bags were heat-sealed and transferred into empty 50 mL serum bottles. Three bottles were used per dietary treatment. Then, 20 mL of the mixture of rumen fluid and buffer was transferred, accompanied by continuous flushing with O_2_-free CO_2_ gas. The bottles were sealed with butyl rubber stoppers and aluminum caps and were incubated on a rotary shaker (JSSI-300 T, JS Research Inc., Gongju, Korea) at 20 r/min for 12 h at 39 °C. After incubation for 6 h, the gas production was measured, and elution buffer and LyJH307 solution pre-flushed with O_2_-free CO_2_ gas were injected into each treatment, and the remaining gas was removed. Gas production was measured at 6, 9, and 12 h using a pressure transducer (Sun Bee Instrument Inc., Seoul, Korea) as described by Theodorou, Williams, Dhanoa, McAllan and France [25].

After measuring the final gas production, the bottle caps were removed, and the bottles were fixed immediately on ice to stop the fermentation. The nylon bags were then removed from the bottles and rinsed under flowing water until the rinsed water ran clear. The washed bags were dried at 60 °C for 72 h and weighed to measure the* in vitro* DM digestibility (IVDMD). Sample fluid (5 mL) was centrifuged at 20,000×*g* for 20 min at 4 °C, the supernatant was discarded, and the pellet was stored at − 80 °C until rumen microbial population analysis. The remaining culture fluid was then transferred to a centrifugal tube for centrifugation at 3,500 r/min and 4 °C for 20 min. The supernatant was collected for the determination of pH and VFA and ammonia-nitrogen (NH_3_-N) concentration. The pH of the culture fluid was measured with a pH meter (FP20, Mettler Toledo, OH, USA). The supernatant for the VFA analysis was acidified with 200 μL of 25% meta-phosphoric acid, and the supernatant for the NH_3_-N analysis was acidified with 200 μL of 0.2 mol/L sulfuric acid, and both were stored at − 20 °C until subsequent VFA and NH_3_-N analyses.

For VFA analysis, 200 μL of the supernatant was diluted with 800 μL of ethyl alcohol anhydrous (4023–2304, Daejung Chemicals, Siheung, Korea) after 20 min of centrifugation at 20,000×*g*. VFA was measured with gas chromatography (Agilent 7890A, Agilent Technology, CA, USA) equipped with a flame ionization detector and capillary column (Nukol™ Fused silica capillary column, 30 m × 250 μm × 0.25 μm, Supelco Inc., PA, USA). The temperature of the oven, injector, and detector was set at 90 °C, 90–200 °C, and 230 °C, respectively. Nitrogen was used as the carrier gas at a flow rate of 30 mL/min. The NH_3_-N concentration was analyzed with several modifications [26]. A 2 μL sample of the supernatant was mixed with 100 μL of phenol color reagent (50 g of phenol, 0.25 g of sodium nitroferricyanide, and 1 L of distilled water) and alkali hypochlorite (25 g of sodium hydroxide, 16.8 mL of sodium hydroxide, and 1 L of distilled water) after the samples were centrifuged at 20,000×*g* for 20 min at 4 °C. The mixture was then incubated in a water bath at 37 °C for 15 min. The NH_3_-N concentration was determined by measuring the optical density at 630 nm by using a microplate reader (iMARK, Bio-Rad Laboratories, Inc., CA, USA).

### DNA extraction

Total DNA was extracted from the pellet stored at − 80 °C by using the repeated bead beating plus column (RBB + C) method [27]. Genomic DNA was treated with RNase A and proteinase K and purified using columns from the DokDo-Prep Genomic DNA Kit (Elpis-Biotech, Daejeon, Korea). The total DNA concentration and purity were measured using a NanoDrop (ND-1000, Thermo Fisher, MA, USA). The purified DNA was stored at − 20 °C until amplicon sequencing and real-time polymerase chain reaction (PCR).

### Quantitative real-time PCR

To assess the absolute abundance of rumen microbes in each sample, quantitative real-time PCR assays were performed on a CFX 96 Touch system (Bio-Rad Laboratories, Inc.) by using several primer sets described in Table 1, as performed in our previous studies [31].

**Table 1:** PCR primers used in quantitative real-time PCR

Target species	Primer	Sequence (5’ → 3’)	Size, bp	Efficiency^a^	References
General bacteria	F	CGGCAACGAGCGCAACCC	130	1.975	[28]
	R	CCATTGTAGCACGTGTGTAGCC			
Ciliate protozoa	F	GCTTTCGWTGGTAGTGTATT	223	1.872	[29]
	R	CTTGCCCTCYAATCGTWCT			
	R	CCCATCCTATAGCGGTAAACCTTTG			
*Streptococcus bovis*	F	TTCCTAGAGATAGGAAGTTTCTTCGG	127	1.944	[30]
	R	ATGATGGCAACTAACAATAGGGGT			

*bp* Base pair

^a^ Efficiency is calculated as [10^–1/slope^]

### PCR amplification and sequencing

PCR amplification was performed using fusion primers targeting the V3 to V4 regions of the 16S rRNA gene with the extracted DNA. For bacterial amplification, the fusion primers used were 341F (5’-AATGATACGGCGACCACCGAGATCTACAC-XXXXXXXX-TCGTCGGCAGCGTC-AGATGTGTATAAGAGACAG-CCTACGGGNGGCWGCAG-3’; underlined sequence indicates the target region primer) and 805R (5’-CAAGCAGAAGACGGCATACGAGAT-XXXXXXXX-GTCTCGTGGGCTCGG-AGATGTGTATAAGAGACAG-GACTACHVGGGTATCTAATCC-3’). The fusion primers were constructed in the following order: P5 (P7) graft binding, i5 (i7) index, Nextera consensus, sequencing adaptor, and target region sequence. The amplifications were performed under the following conditions: initial denaturation at 95 °C for 3 min, followed by 25 cycles of denaturation at 95 °C for 30 s; primer annealing at 55 °C for 30 s; and extension at 72 °C for 30 s, with a final elongation at 72 °C for 5 min. The PCR product was confirmed using 1% agarose gel electrophoresis and visualized under a Gel Doc system (Bio-Rad). The amplified products were purified with CleanPCR (CleanNA). Equal concentrations of purified products were pooled together, and short fragments (non-target products) were removed with CleanPCR (CleanNA). The quality and product size were assessed on a Bioanalyzer 2100 (Agilent, Palo Alto, CA, USA) using a DNA 7500 chip. Mixed amplicons were pooled and the sequencing was undertaken at Chunlab, Inc. (Seoul, Korea), with an Illumina MiSeq Sequencing System (Illumina, USA) according to the manufacturer’s instructions.

### Data analysis pipeline

To analyze the 16S rRNA gene amplicon sequences, the sequencing data were analyzed using Quantitative Insights into Microbial Ecology 2 (QIIME2) [32]. Briefly, after demultiplexing, the primer sequences were removed using FASTX-Toolkit [33]. The sequence reads were quality-filtered, denoized, and the paired-end sequence data were merged using FLASH2 [34]. The chimeric sequences were then removed using the DADA2 plugin, and amplicon sequencing variants (ASVs) were taxonomically classified from the Silva (SSU138) 16S rRNA gene database [35]. Sequences classified as mitochondria, chloroplast, and unassigned ASVs were removed from the dataset. The alpha diversities (Shannon’s index, Simpson’s index, Chao1 estimates, and evenness) were calculated from rarefied abundance tables using 14,673 sequences per sample. To investigate the dissimilarity of overall microbiota between treatments, principal coordinate analysis (PCoA) based on Bray-Curtis dissimilarity and weighted UniFrac distance matrices were used. The functional metagenomic predictions were generated using a PICRUSt2 algorithm (version 2.3.0-b) [36].

### Statistical analysis

All data were checked for normal distribution by using the Shapiro-Wilk test in SAS 9.4 (SAS Institute Inc., NC, USA). Normally distributed data including all alpha diversity measurements (Shannon’s index, Simpson’s index, Chao1 estimates, and evenness), rumen fermentation data (gas production, IVDMD, NH_3_-N, VFA, and lactate), and various functional features predicted from the 16S rRNA gene using five different reference databases (KEGG, Pfam, clusters of orthologous genes [COG], enzyme classification, and MetaCyc) were further analyzed using t-tests in SAS 9.4. Statistical significance for normally distributed data was declared at *P* < 0.05, and a trend was speculated at 0.05 ≤ *P* ≤ 0.10. For analyzing treatment, time, and interaction effect on gas production, two-way analysis of variance was performed. The PCoA results of beta diversity were analyzed by permutational multivariate analysis of variance (PERMANOVA) to assess if microbial community significantly differed between treatments. A major microbiome was defined as those ASVs present in greater than 50% of the samples. The differences in major microbiome and functional features (KEGG pathway and module) were analyzed by the linear discriminant analysis effect size (LEfSe), which implements a non-parametric Kruskal-Wallis sum-rank test followed by linear discriminate analysis. Spearman’s rank correlations between the relative abundance of differentially abundant prokaryotic taxa revealed by LEfSe and fermentation data were analyzed using PROC CORR procedures of SAS 9.4 and visualized using the R package corrplot (v. 3.6.3).

## Results

### Effect of endolysin LyJH307 on ruminal fermentation characteristics and qPCR

Compared to CON, endolysin LyJH307 supplementation did not affect gas production at each time points (Table 2). However, the overall gas production had shown not only time effect (*P* < 0.0001), but also treatment effect (*P* = 0.0132). The pH was significantly higher in the LyJH307 group than in the CON group (*P* = 0.0335), whereas IVDMD, NH_3_-N, and total VFA production were not changed by endolysin LyJH307 supplementation (IVDMD, *P* = 0.3597; NH_3_-N, *P* = 0.1485; and total VFA, *P* = 0.2592). In each proportion of individual VFAs, the proportion of acetate was significantly higher in the LyJH307 group than in the CON group (*P* = 0.0362), whereas that of propionate was significantly lower in the LyJH307 group (*P* = 0.0379), thereby increasing the acetate to propionate ratio (A:P ratio) in LyJH307 (*P* = 0.0291). However, endolysin LyJH307 supplementation did not affect the proportion of butyrate, iso-butyrate, valerate, and iso-valerate (butyrate, *P* = 0.2435; iso-butyrate, *P* = 0.3502; valerate, *P* = 0.2752; and iso-valerate, *P* = 0.3502). Endolysin LyJH307 supplementation significantly increased only the D-lactate concentration compared to the CON group (*P* = 0.0340) and total lactate concentration was high in the LyJH307 group (*P* = 0.0824).

**Table 2:** Gas production and rumen fermentation parameters by endolysin LyJH307 supplementation in an* in vitro* experiment at 12 h of incubation

	Treatments^1^			
Items^a^	CON	LyJH307	SEM	*P*-value
Gas^2^, mL/g DM				
6 h	52.2	50.0	1.10	0.12
9 h	87.3	82.7	1.91	0.11
12 h	106	103	2.2	0.25
IVDMD, %	40.1	38.7	1.16	0.36
NH_3_-N, mg/100 mL	7.87	9.21	0.553	0.15
pH	5.57	5.69	0.033	0.034
Total VFA, mmol/L	83.1	89.1	4.39	0.26
Acetate, mmol/mol	428	448	6.4	0.036
Propionate, mmol/mol	342	325	4.4	0.038
Butyrate, mmol/mol	200	195	3.0	0.24
Iso-butyrate, mmol/mol	1.96	2.16	0.171	0.35
Valerate, mmol/mol	22.2	20.9	0.80	0.28
Iso-valerate, mmol/mol	6.96	8.12	1.726	0.63
A:P ratio	1.25	1.38	0.033	0.029
Total lactate, mmol/L	16.8	18.9	0.87	0.082
D-lactate, mmol/L	9.74	11.26	0.404	0.034
L-lactate, mmol/L	7.02	7.63	0.642	0.46

^a^ *SEM* Standard error of the mean, *DM* Dry matter, *IVDMD* *In vitro* dry matter digestibility, *NH*_*3*_*-N* Ammonia-nitrogen, *VFA* Volatile fatty acids, *A:P ratio* Acetate to propionate ratio

^1^ *CON*, corn grain with elution buffer of the same volume of endolysin treatment; *LyJH307*, corn grain with endolysin LyJH307 (4 U/mL)

^2^ Treatment effect, *P* = 0.0114; Time effect, *P* < 0.0001; Interaction, *P* = 0.9353

The absolute abundance of total bacteria and ciliate protozoa did not show any significant differences between treatments (Table 3, total bacteria, *P* = 0.6036; ciliate protozoa, *P* = 0.8271), whereas the absolute abundance of *S. bovis* was significantly decreased by endolysin LyJH307 supplementation (*P* < 0.0289).

**Table 3:** Microbial absolute abundances by endolysin LyJH307 supplementation in an *in vitro* experiment at 12 h of incubation

	Treatments^b^			
Items^a^	CON	LyJH307	SEM	*P*-value
Total bacteria^c^	3.82	3.68	0.231	0.60
Ciliate protozoa^d^	1.37	1.43	0.206	0.83
*Streptococcus bovis*^d^	5.70^a^	1.76^b^	1.161	0.029

^a^ *SEM*, standard error of the mean

^b^ *CON*, corn grain with elution buffer of the same volume of endolysin treatment; *LyJH307*, corn grain with endolysin LyJH307 (4 U/mL)

^c^ × 10^10^ copies/mL of rumen fluid

^d^ × 10^8^ copies/mL of rumen fluid

### Effect of endolysin LyJH307 on the rumen microbiota

**Fig. 1:**
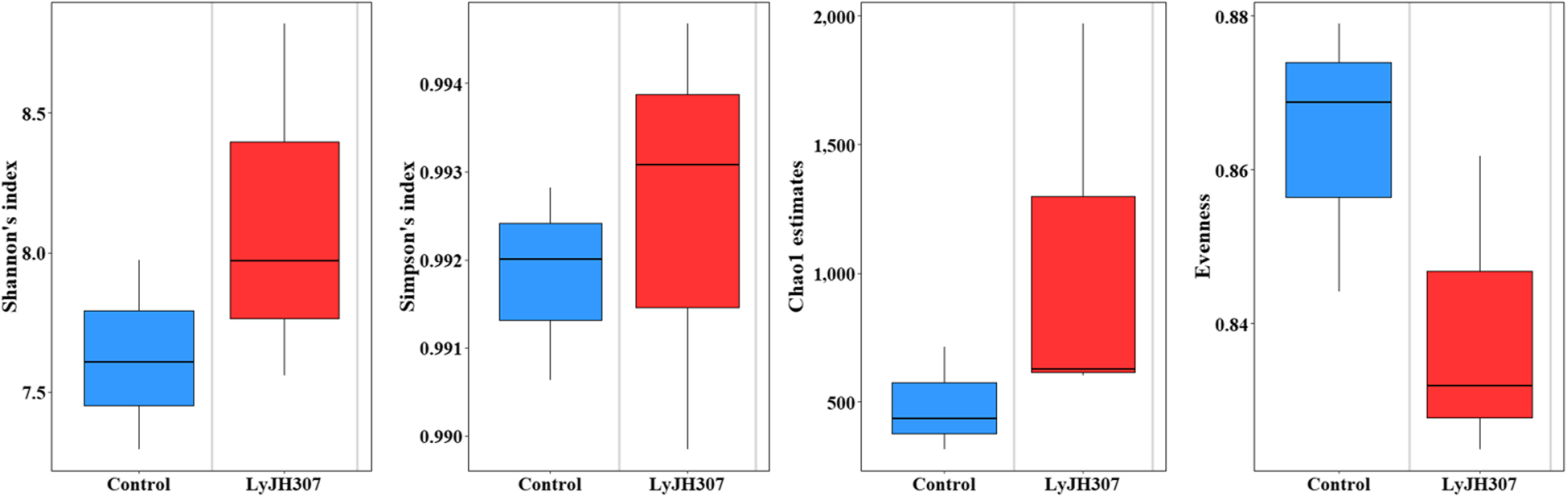
Alpha diversity indices after endolysin LyJH307 supplementation in an in vitro experiment at 12 h of incubation

**Fig. 2:**
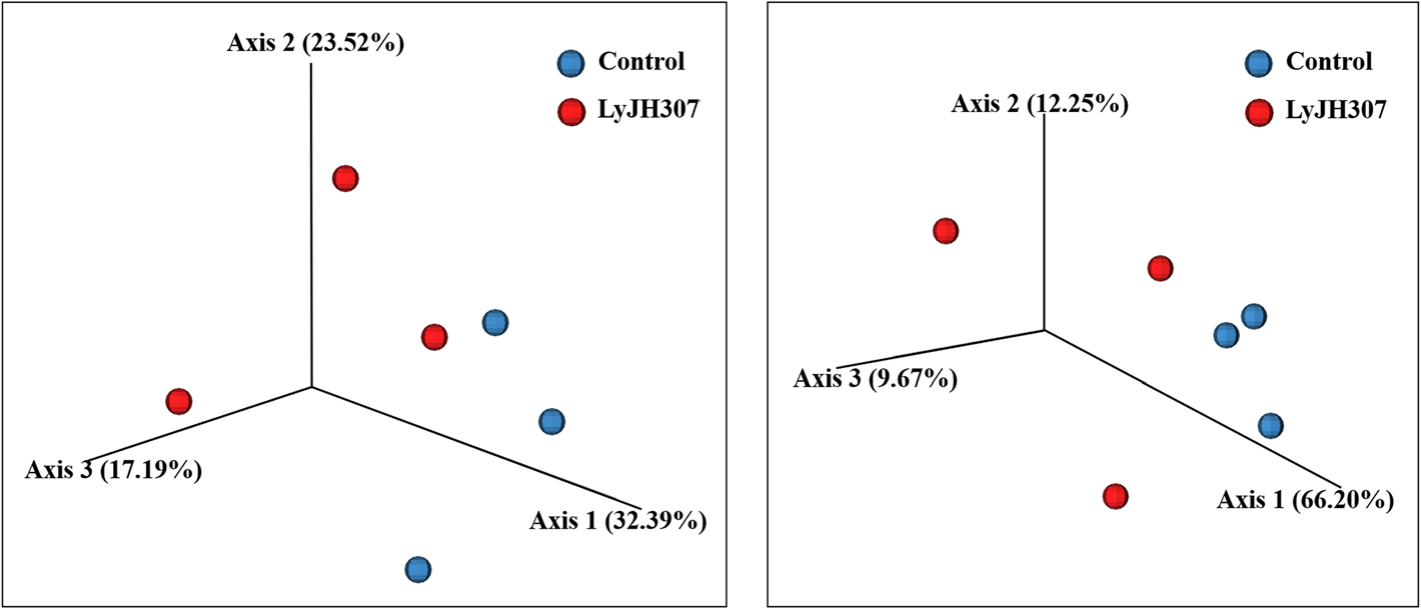
Principal coordinates analysis based on (**A**) Bray-Curtis dissimilarity and (**B**) weighted UniFrac distances. PERMANOVA analysis was used for comparing differences between the control and LyJH307 groups

In the present study, 260,866 sequences were obtained by the 16S rRNA sequence analysis with an average of 43,477 ± 12,838 sequences per sample. Through quality filtering by QIIME2 (Q score > 20), 148,786 sequences (57% of the raw reads) were generated with an average of 24,797 ± 9,238 sequences per sample. There were no significant differences in the alpha diversity measurements, including Shannon’s index, Simpson’s index, Chao1 estimates, and evenness (Fig. 1, Shannon’s index, *P* = 0.5127; Simpson’s index, *P* = 0.5127; Chao1 estimates, *P* = 0.2752; and evenness, *P* = 0.1266)). For the beta diversity, we presented two PCoA results based on Bray-Curtis dissimilarity and weighted UniFrac distance, followed by PERMANOVA analysis (Fig. 2). The PCoA result based on Bray-Curtis dissimilarity showed statistical tendency between the CON and LyJH307 groups (Fig. 2A, *P =* 0.0970), whereas there was no significant change in the PCoA result based on the weighted UniFrac distances (Fig. 2B, *P* = 0.5030).

**Fig. 3:**
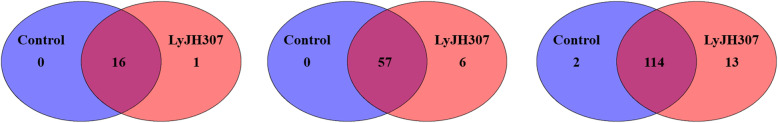
Venn diagram of commonly and exclusively identified prokaryotic taxa in collapsed BIOM table for **A** phylum, **B** family, and **C** genus levels

In the Venn diagram made by the major microbiome data, 16 of 17 phyla were shared, and 57 of 63 families were shared between the CON and LyJH307 groups. At the phylum and family levels, specific taxa were observed only in the LyJH307 group (Fig. 3A and B). In addition, 114 of 129 genera were shared, and specific taxa in the LyJH307 group were higher than those in the CON group (Fig. 3C).

**Fig. 4:**
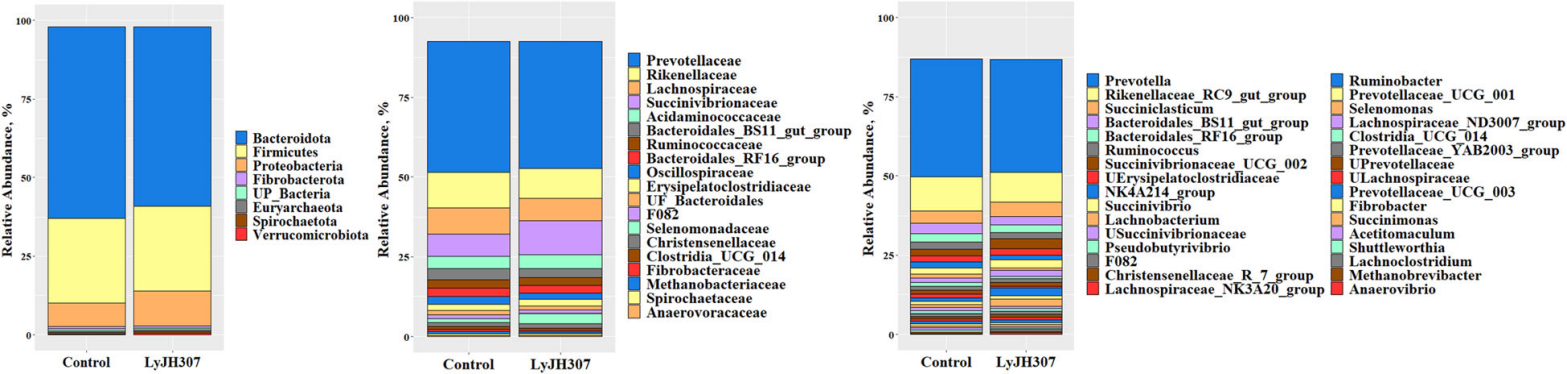
Distribution of the predominant rumen microbiota at **A** phylum, **B** family, and **C** genus levels with each proportion above 0.5% and found in at least 50% the samples in each treatment

The predominant taxa with relative abundance above 0.5% in at least one group are shown in Fig. 4. At the phylum level, eight phyla had a relative abundance > 0.5%, and Bacteroidota (CON, 60.9% vs. LyJH307, 56.7%), Firmicutes (CON, 27.2% vs. LyJH307, 27.2%), and Proteobacteria (CON, 7.3% vs. LyJH307, 11.2%) were the three dominant phyla (Fig. 4A), while the other phyla had a relatively minor proportion (both treatments, relative abundance < 1%). A total of 19 families were the dominant taxa that had a relative abundance of > 0.5% (Fig. 4B), and there were no significant differences in major families between the CON and LyJH307 groups. At the genus level, the three dominant genera in the CON group were Prevotellaceae (41.1%), Rikenellaceae (11.1%), and Lachnospiraceae (8.3%), whereas the three dominant genera in the LyJH307 group were Prevotellaceae (39.8%), Succinivibrionaceae (10.7%), and Rikenellaceae (9.3%) (Fig. 4C).

**Fig. 5:**
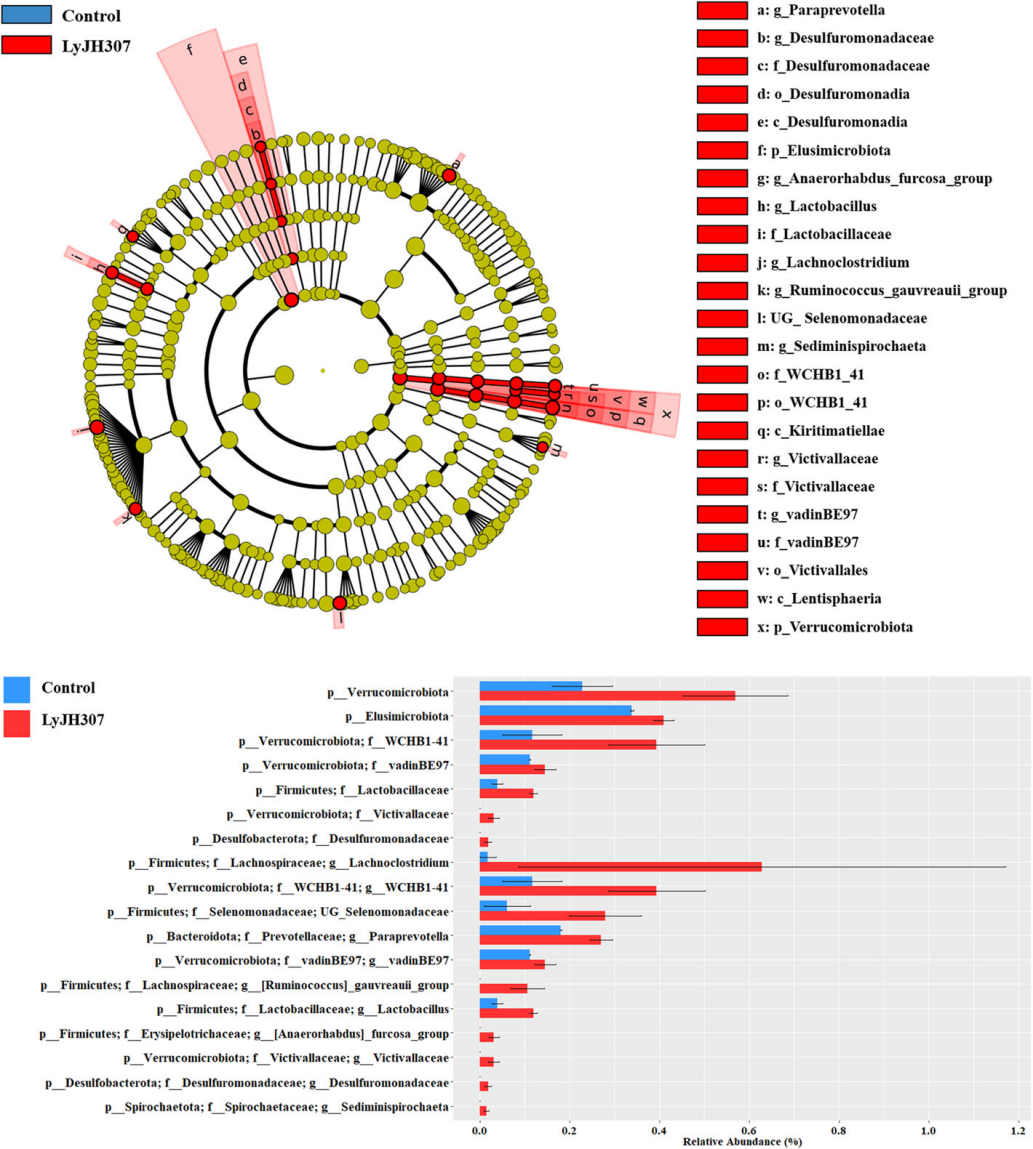
Differentially abundant microbial taxa displayed by **A** cladogram and **B** bar plot at the genus level affected by endolysin LyJH307 supplementation detected using linear discriminant analysis (LDA) effect size (LEfSe) with a LDF score ≥ 2

The differentially abundant taxa at the phylum, family, and genus levels between the CON and LyJH307 groups were identified using LEfSe (Fig. 5). At the phylum level, the relative abundance of Elusimicrobiota and Verrucomicrobiota were significantly higher in the LyJH307 group than in the CON group (Fig. 5). At the family level, LyJH307 supplementation significantly increased the relative abundance of three genera within Verrucomicrobiota (WCHB1–41, vadinBE97, and Victivallaceae), one genus within Firmicutes (Lactobacillaceae), and Desulfuromonadaceae (Fig. 5B). At the genus level, a total of 11 genera including *Lachnoclostridium*, *WCHB*1–41, unclassified genus Selenomonadaceae (UG_Selenomonadaceae), *Paraprevotella*, *vadinBE*97, *Ruminococcus gauvreauii* group, *Lactobacillus*, *Anaerorhabdus furcosa* group, *Victivallaceae*, *Desulfuromonadaceae*, and *Sediminispirochaeta* had significantly higher relative abundance in the LyJH307 group than in the CON group; thus, there were no genera enriched in the CON group (Fig. 5B). Among the differentially abundant genera, several genera were only detected in the LyJH307 group, including *Ruminococcus gauvreauii* group, *Anaerorhabdus furcosa* group, *Victivallaceae*, *Desulfuromonadaceae*, and *Sediminispirochaeta*.

### Effect of endolysin LyJH307 on the predicted functions of the microbiota

**Fig. 6:**
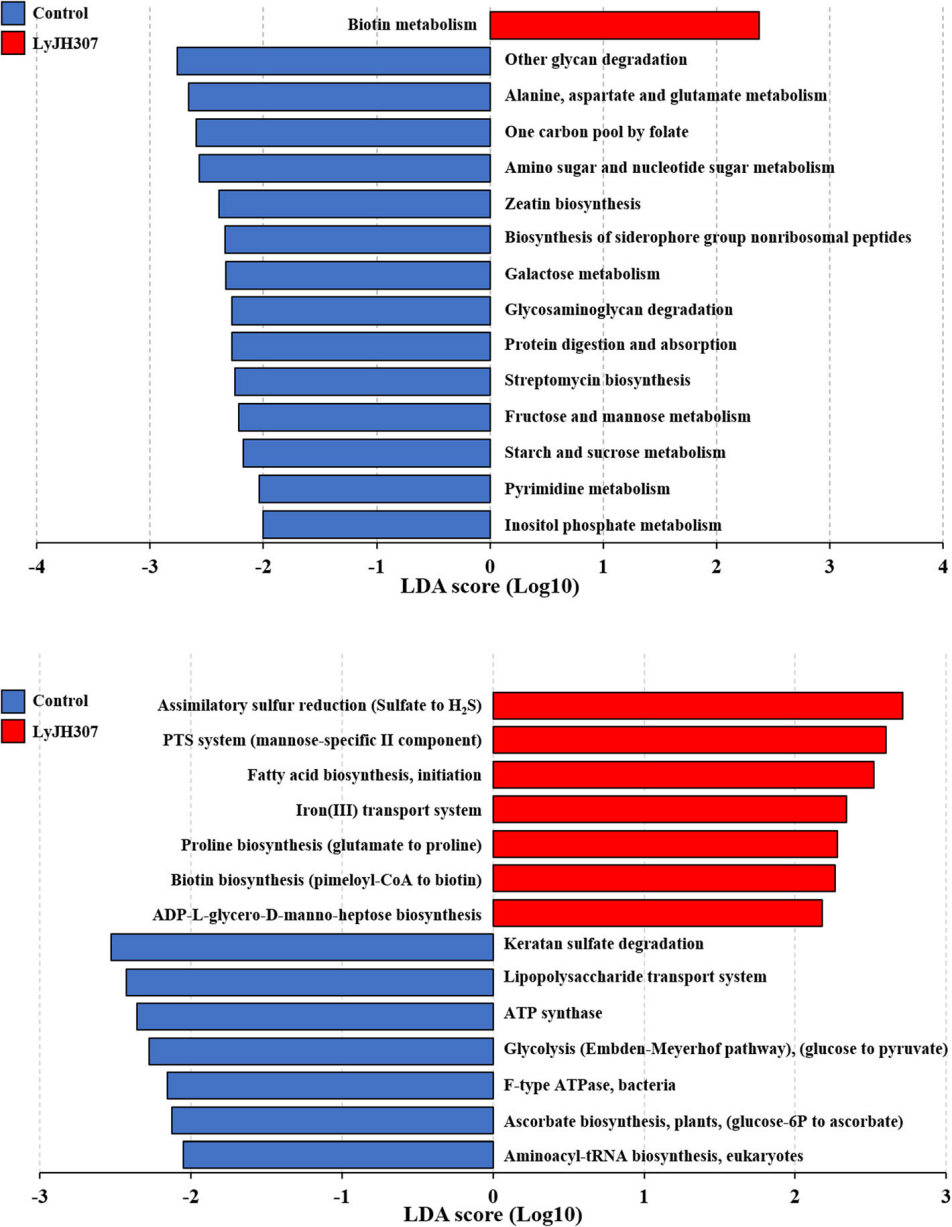
Differentially abundant predicted functional features of Kyoto Encyclopedia of Genes and Genomes (KEGG) pathways and modules affected by endolysin LyJH307 supplementation detected using linear discriminant analysis effect size (LEfSe) with a linear discriminant analysis effect size ≥2

To identify the functional changes of ruminal bacteria metabolism by LyJH307 supplementation, the functional composition profiles were predicted from 16S rRNA gene sequencing data with PICRUSt2, and the various functional features were predicted from the 16S rRNA gene using five different reference databases (KEGG, Pfam, COG, enzyme classification, and MetaCyc) (Table 4). LyJH307 affected the overall increase in the numbers of total functional features (KEGG orthologs, *P* = 0.0559; KEGG modules, *P* = 0.0535; Pfam, *P* = 0.0302; COG, *P* = 0.0566; and MetaCyc pathways *P* = 0.0969) at least showing statistical tendency (Table 4). However, the overall predicted functional features in the KEGG pathways were enriched in the CON group, with 14 KEGG pathways (other glycan degradation, ko00511; alanine, aspartate, and glutamate metabolism, ko00250; one carbon pool by folate, ko00670; amino sugar and nucleotide sugar metabolism, ko00520; zeatin biosynthesis, ko00908; biosynthesis of siderophore group nonribosomal peptides, ko01053; galactose metabolism, ko00052; glycosaminoglycan degradation, ko00531; protein digestion and absorption, ko04974; streptomycin biosynthesis, ko00521; fructose and mannose metabolism, ko00051; starch and sucrose metabolism, ko00500; pyrimidine metabolism, ko00240; and inositol phosphate metabolism, ko00562), except for biotin metabolism (ko00780) that was higher in the LyJH307 group (Fig. 6A). In the KEGG modules, the predicted functional features were found to be enriched differently between the CON and LyJH307 groups (Fig. 6B). In the CON group, keratan sulfate degradation (M00079), lipopolysaccharide transport system (M00250), ATP synthase (M00164), glycolysis (M00001), F-type ATPase (M00157), ascorbate biosynthesis (M00114), and aminoacyl-tRNA biosynthesis (M00359) were higher in the KEGG modules, whereas assimilatory sulfur reduction (M00176), PTS system (M00276), fatty acid biosynthesis (M00082), iron (III) transport system (M00190), proline biosynthesis (M00015), biotin biosynthesis (M00123), and ADP-L-glycero-D-manno-heptose biosynthesis (M00064) were higher in the LyJH307 group (Fig. 6B). Despite the changes in the low hierarchical level (KEGG pathways and modules), only two features, the biosynthesis of other secondary metabolites (*P* = 0.0809) and nucleotide metabolism (*P* = 0.0809), decreased by endolysin LyJH307 supplementation among the top 20 KEGG level 2 (Supplemental Table 1).

**Table 4:** Number of predicted functional features including KEGG hierarchies (orthologs, modules, and pathways), enzyme classification, MetaCyc pathways, Pfam, and clusters of orthologous genes by LyJH307 supplementation in an in vitro experiment at 12 h of incubation

	Treatments^b^			
Items^a^	CON	LyJH307	SEM	*P*-value
KEGG orthologs	5137	5354	60.3	0.056
KEGG modules	270	277	2.1	0.054
KEGG pathways	137	142	3.2	0.24
Pfam	5954	6254	75.5	0.030
COG	3887	4007	36.2	0.057
EC	1603	1656	21.8	0.14
MetaCyc pathways	328	339	3.8	0.097

^a^ *SEM* Standard error of the mean, *KEGG* Kyoto Encyclopedia of Genes and Genomes, *COG* Clusters of orthologous genes, *EC* Enzyme classification

^b^ CON, corn grain with elution buffer of the same volume of endolysin treatment; LyJH307, corn grain with recombinant LyJH307 (4 U/mL)

### Correlations between microbial taxa and ruminal fermentation characteristics

**Fig. 7:**
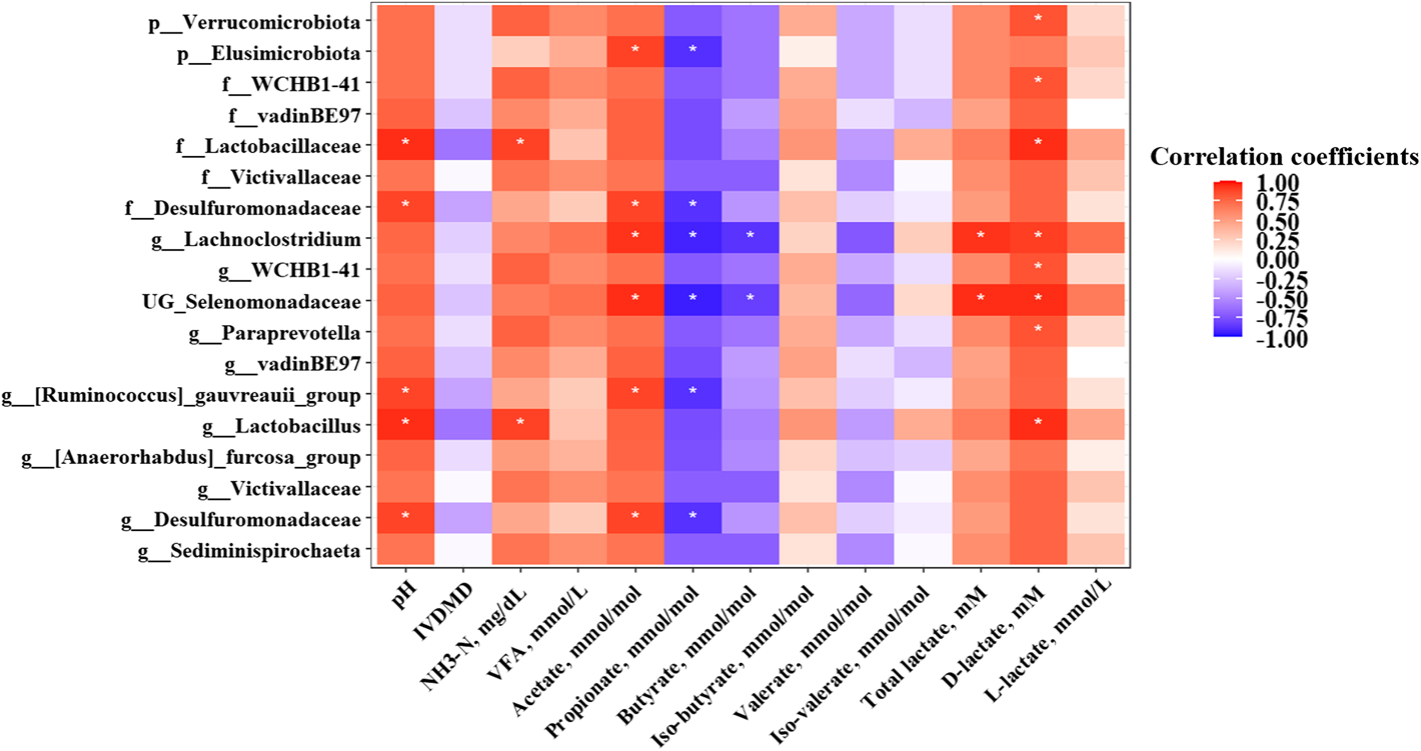
Spearman’s correlation coefficients between rumen fermentation characteristics and the relative abundance of differentially abundant prokaryotic taxa at phylum, family, and genus levels by the supplementation of endolysin LyJH307 in an *in* *vitro* experiment. Positive correlation (closer to 1) is shown as red, and negative correlation (closer to −1) is shown as blue. Asterisk indicates significant correlations between rumen fermentation characteristics and the relative abundance of taxa (|r| ≥ 0.8 and *P* < 0.05)

Strong correlations (|*r*| > 0.8, *P* < 0.05) between ruminal fermentation characteristics and differently abundant taxa analyzed by LEfSe were detected (Fig. 7). Lactobacillaceae, Desulfuromonadaceae (family), *Ruminococcus gauvreauii* group, *Lactobacillus*, and *Desulfuromonadaceae* (genus), all of which were higher in the LyJH307 group than in the CON group, were positively correlated with ruminal pH. In the individual VFA proportions, Elusimicrobiota, Desulfuromonadaceae (family), *Lachnoclostridium*, unclassified genus (UG)_Selenomonadaceae, *Ruminococcus gauvreauii* group, and *Desulfuromonadaceae* (genus) were all increased in the LyJH307 group and were positively correlated with the acetate proportion and negatively correlated with that of propionate. Verrucomicrobiota, WCHB1–41, Lactobacillaceae, *Lachnoclostridium*, WCHB1–41, UG_Selenomonadaceae, *Paraprevotella*, and *Lactobacillus* were positively correlated with D-lactate concentration.

## Discussion

The rumen is a distinct digestive organ that degrades plant-originated nutrients that are non-edible to mono-gastric animals and symbiotic ecosystem-living anaerobic bacteria, archaea, protozoa, and fungi sharing fermentation metabolites [37]. Therefore, artificial modulation of rumen fermentation is challenging. Previously, several studies observed that supplementation with ionophore antibiotics could decrease the ruminal bacteria (especially gram-positive bacteria) in a high concentrate diet, thereby preventing a severe decrease of ruminal pH [38–40]. However, the potential for antibiotic resistance in ruminal bacteria has made it impossible to use ionophore antibiotics as a feed additive to modulate rumen fermentation [41, 42]. The present study is the first trial presenting a new strategy to control *S. bovis* specifically using endolysin. This study confirmed that endolysin LyJH307 supplementation decreased the absolute abundance of *S. bovis* efficiently (approximately 70%, compared to CON group), thereby increasing ruminal pH in an *in vitro* batch culture.

Previously, we observed that the same dosage of endolysin LyJH307 used in the present study did not significantly change the absolute abundance of *S. bovis* and rumen fermentation parameters (including pH, IVDMD, and VFA) when the endolysin LyJH307 was added at the start of the incubation in an *in vitro* system (data not shown). We assumed that the reason why the endolysin was not working at initial incubation time was due to the insufficient abundance of *S. bovis*. In the rumen, protein sources can be degraded by various bacteria such as *P*. *ruminicola*, *S*. *ruminantium*, *Butyrivibrio fibrisolvens*, *Clostridium proteoclasticus*, and *Eubacterium ruminantium* [43]. Besides, the solubility of proteins is one of the key factors for determining the degradability of protein [44]. The endolysin LyJH307 has a high solubility among a wide range of pH (5.0–6.5), temperature (25–55 °C), and NaCl concentration (0–500 mmol/L). Therefore, the endolysin might be degraded by ruminal proteolytic bacteria before controlling *S. bovis*. We expected that the supplementation time is important to induce adequate lytic activity in an *in vitro* system. In the present study, endolysin LyJH307 supplementation at 6 h incubation time markedly decreased the absolute abundance of *S. bovis* (approximately 70%). In addition, the reduction rate of *S. bovis* in the present study was similar to previous results presenting reduction rates of the *S. bovis* group (*S. equinus*, *S. gallolyticus* subspecies *pasteurianus*, *S. alactolyticus*, and *S. infantarius* subspecies *infantarius*) in single cultures [15]. Therefore, it was assumed that the application of endolysin to control specific ruminal bacteria was a possible strategy, but the supplementation timing or frequency should be considered to use endolysin as a rumen microbial modifier.

Several agents to inhibit ruminal bacteria were evaluated in an *in vitro* system, and the ability to change the rumen microbiota and rumen fermentation has been shown [45–47]. Shen, Liu, Yu (47) reported that monensin and nisin supplementation decreased the absolute abundance of total bacteria and overall rumen fermentation (gas and VFA production) with a concomitant change in the major rumen microbiota. In the present study, endolysin LyJH307 did not affect the absolute abundance of total bacteria, ciliate protozoa (Table 3) and major microbiota from phylum to genus levels (having relative proportions > 1%) (Fig. 4). *Prevotella* was the most abundant genus of Bacteroidota and *Succiniclasticum* was that of Firmicutes in the present study. *Prevotella* is the most predominant ruminal genus [48], and many are related to the digestion of polysaccharides and protein [49]. *Succiniclasticum* is known for its role in converting succinate to propionate [50], and previous studies have shown that the relative proportion of *Succiniclasticum* can be increased in a high grain diet, in agreeance with our findings [51, 52]. The relative proportion of Verrucomicrobiota was higher in animals fed a low grain diet than those fed a high grain diet [53, 54], and rumen under normal pH contained a higher Verrucomicrobiota proportion than those undergoing SARA [55]. In the present study, the relative proportion of Verrucomicrobiota was higher in the LyJH307 group, which showed higher ruminal pH compared to that of the CON. Therefore, we postulated that Verrucomicrobiota might be used as one of the potential biomarkers indicating the SARA condition. In the minor taxa at the genus level, 11 genera with a relative proportion < 0.1% were increased by endolysin LyJH307, including *Lachnoclostridium*, WCHB1–41, UG_Selenomonadaceae, *Paraprevotella*, vadinBE97, *Ruminococcus gauvreauii* group, *Lactobacillus*, *Anaerohabdus furcosa* group, *Victivallaceae*, *Desulfuromonadaceae*, and *Sediminispirochaeta*. In a previous study, endolysin LyJH307 had a narrow range of lytic activity, specifically in the *S. bovis* group [15]. The narrow specificity of LyJH307 to kill target bacteria might lead to an increase of minor microbial taxa competing with *S. bovis* to use starch carbohydrates; therefore, no significant changes in rumen fermentation (gas, VFA, and IVDMD) were observed.

In the present study, endolysin LyJH307 induced an increase in acetate proportion but a decrease in propionate proportion, thus increasing the A:P ratio. The increased proportion of acetate in the LyJH307 group can be explained by the increase of several minor bacterial genera, such as *Lachnoclostridium*, *Paraprevotella*, *Ruminococcus gauvreauii* group, and *Sediminispirochaeta*. The *Lachnoclostridium* and *Ruminococcus gauvreauii* group are gram-positive and obligate anaerobic bacteria that are known for producing acetate as the main end-product of glucose fermentation [56, 57]. Previously, *Paraprevotella* and *Sediminispirochaeta* were reported to produce acetate as the end product of glucose metabolism [58, 59]. Thus, the reduction of *S. bovis* by supplementation of endolysin LyJH307 might induce the increase of several minor genera producing acetate as the main fermentation product. *Lachnoclostridium*, UG_Selenomonadaceae, *Ruminococcus gauvreauii*_group, and *Desulfuromonadaceae* (genus) that were higher in the LyJH307 group than in the CON group were positively correlated with acetate proportion and negatively correlated with propionate proportion. Little is known about the function of UG_Selenomonadaceae and *Desulfuromonadaceae* (genus) in producing acetate in the rumen, therefore further research is required to determine their relationship with acetate and propionate proportion.

*S. bovis* produces lactate as the major fermentation product when the starch source is enriched in the diet [60], and this bacterial species can generate more ATP per hour than the other ruminal amylolytic bacteria [61]. Therefore, an increase of ruminal pH by the supplementation of LyJH307 was expected. One of the standard points of ruminal pH for SARA is below 5.6 and that of ruminal pH for acute acidosis is below 5.0 [1]. The addition of LyJH307 significantly increased the ruminal pH, although the range of ruminal pH in both treatments was close to SARA conditions (CON group, 5.57 vs. LyJH307 group, 5.69). The rumen epithelial tissue absorbs the ruminal fermentation production, thereby stabilizing the ruminal pH and improving animal productivity. Considering that the increased ruminal pH by LyJH307 in an in vitro system which is an enclosed condition, the effect of pH stabilization of LyJH307 through specific inhibition of *S. bovis* might be potent in an *in vivo* condition. Further research will be needed to prove the effect of LyJH307 on pH stabilization in an *in vivo* condition, because the outcomes from in vitro batch culture experiment are not always linked with *in vivo* condition.

In the present study, endolysin LyJH307 supplementation increased D-lactate concentration with a concomitant increase in total lactate production. *Lactobacillus* is generally connected with acute acidosis and SARA because of its ability to produce lactate as the main fermentation product and high acid resistance [1]. *Lactobacillus* can digest starch sources including corn and barley under non-acidosis conditions [62]. Therefore, the increased proportion of *Lactobacillus* in the LyJH307 group might be related to the increased availability of a starch source achieved by decreasing the abundance of *S. bovis*. Previous studies have shown that several species of *Selenomonas* can produce lactate as the main fermentation product with high concentrations of glucose, whereas the major fermentation products are changed to acetate and propionate in a glucose-limited condition [63–65]. In the present study, considering that the feed ingredient was only corn grain, it was speculated that the diet might affect the alteration in the major fermentation product of UG_Selenomonadaceae to lactate, especially affecting an increase of D-lactate concentration. In addition, *Lachnoclostridium*, WCHB1–41, UG_Selenomonadaceae, *Paraprevotella*, and *Lactobacillus,* which were increased in the LyJH307 group, were positively correlated with D-lactate concentration, therefore they might have also contributed to the high D-lactate concentration in the LyJH307 group. However, since correlation does not mean causation between factors, further research is required to determine their relationship with D-lactate production.

The alteration of ruminal microbiota composition by using dietary feed additives is linked with a change of microbial functional features. In the present study, PICRUSt2 was used to predict the functional changes of ruminal microbiota associated with LyJH307 supplementation. The addition of endolysin LyJH307 altered several bacterial populations that had a minor relative proportion (< 1%) across all samples. In addition to the similar alpha-diversity measurements across the treatments, the number of predicted functional features (KEGG orthologs, KEGG modules, PFAM, COG, and MetaCyc pathways) was tended to be higher in endolysin treatment. However, among the differentially abundant KEGG pathways between the CON and LyJH307 groups analyzed by LEfSe, the KEGG pathways involved in carbohydrate metabolism (e.g., starch and sucrose metabolism, amino sugar and nucleotide sugar metabolism, fructose and mannose metabolism, and galactose metabolism, and inositol phosphate metabolism) were abundant in the CON group. Thus, the addition of endolysin LyJH307 might change carbohydrate metabolism with concomitant changes in minor rumen microbiota, thereby inducing higher ruminal pH in the LyJH307 group than in the CON group.

Previously, Bhatt, Mohapatra, Anand, Kuntal and Mande (66) reported the possibility of incorrect assignments in KEGG modules related to bacterial systems, leading to misinterpretation. Ascorbate biosynthesis plants, (glucose-6-phosphate to ascorbate) (M00114), which was higher in the CON group than in the LyJH307 group, is a plant metabolism pathway related to the formation of L-ascorbate from GDP-D-mannose. KEGG orthologs contained ascorbate biosynthesis, plants, (glucose-6P to ascorbate) in the present study entry were mannose-1-phosphate guanylyltransferase and mannose-6-phosphate isomerase, which are enzymes converting GDP-D-mannose to fructose-6-phosphate. Thus, the module definition of ascorbate biosynthesis, plants, (glucose-6-phosphate to ascorbate) in the present study was misused as previously suggested [66].

F-type ATPase is the most common ATPase/synthase found in bacteria, and it generally acts as ATP synthase driving ATP synthesis through a passive flux of H^+^ in aerobic organisms [67]. Several studies have shown that F-type ATPase can be used for ATP synthase by translocation of H^+^ and Na^+^ in strict anaerobes [68–70]. Lu et al. [71] showed that the F-type ATPase was significantly increased with an increase in dietary grain level in the diet. In the present study, considering that glycolysis (M00001), ATP synthase (M00164), and F-type ATPase (M00157) were higher in the CON group than in the LyJH307 group, the use of endolysin LyJH307 might induce reduction of efficiency in carbohydrate availability by specifically inhibition of *S. bovis*.

This study identified that the lytic activity of LyJH307 against *S. bovis* under *in vitro* fermentation, and possible degradation of endolysin. Besides, the stabilization of ruminal pH and changes of ruminal microbiota related to carbohydrate metabolism by the reduction of *S. bovis* were observed. A major innovation is that we used a new approach to modulate ruminal microorganisms and evaluated the availability of endolysin as a new feed additive. For the application of endolysin LyJH307 in on-farm condition, the strategy to supply endolysin persistently would be needed such as a bolus type feed additive. However, this study has several limitations; (1) we used corn grain as an experimental substrate, (2) we confirmed the effect of endolysin LyJH307 under an in vitro batch culture system. Therefore, future studies should be guaranteed to investigate the effects of endolysin LyJH307 on rumen fermentation under various conditions (e.g. administration methods, type of feeding diets, and in vivo condition).

## Conclusion

In summary, endolysin LyJH307 supplementation markedly decreased the absolute abundance of *S. bovis* and improved the ruminal pH thereby eliminating any negative effects on overall fermentation parameters (gas, IVDMD, and total VFA) in an *in vitro* experiment. In the 16S amplicon sequencing analysis, endolysin LyJH307 caused a shift in minor rumen microbiota and its metabolic pathways related to carbohydrate metabolism, and these changes were associated with the increase of acetate proportion and D-lactate concentration. This study provides the first insight into the availability of endolysin as a specific modulator for rumen and shows the possibility of endolysin degradation by rumen microbiota. These findings provide new perspectives for modulating ruminal digestion and disease causative bacteria by using endolysin in the rumen.

## Supplementary Information

**Additional file 1: Supplemental Table 1.** Relative abundance of predicted KEGG level 2 (Top 20) affected by endolysin LyJH307 in an in vitro experiment at 12 h of incubation.

## Data Availability

The raw 16S rRNA sequences have been deposited in the NCBI SRA under BioProject PRJNA699718. All analyzed microbial datasets in the present study are available from the corresponding author on reasonable request.

## Abbreviations

aNDF: Neutral detergent fiber
ASVs: Amplicon sequencing variants
A:P ratio: Acetate to propionate ratio
CBD: C-terminal cell wall binding domain.
CON: Control diet
CP: Crude protein
DM: Dry matter
EAD: N-terminal enzymatically active domain
IVDMD: In vitro dry matter digestibility
KEGG: Kyoto Encyclopedia of Genes and Genomes
LEfSe: Linear discriminant analysis effect size
LyJH307: Endolysin LyJH307 treatment
NH_3_-N: Ammonia-nitrogen
NFC: Non-fiber carbohydrate
PERMANOVA: Permutation multivariate analysis of variance
PG: Peptidoglycan
PCoA: Principal coordinate analysis
QIIME2: Quantitative Insights into Microbial Ecology 2
SARA: Subacute ruminal acidosis
VFA: Volatile fatty acids
