# Supplementary material for: Specific inhibition of Streptococcus bovis by endolysin LyJH307 supplementation shifts the rumen microbiota and metabolic pathways related to carbohydrate metabolism
Source: J Anim Sci Biotechnol. 2021 Aug 4;12:93. doi: 10.1186/s40104-021-00614-x (PMC8335910; doi:10.1186/s40104-021-00614-x)
Supplement: Supplementary file 1 — Additional file 1: Supplemental Table 1. Relative abundance of predicted KEGG level 2 (Top 20) affected by endolysin LyJH307 in an in vitro experiment at 12 h of incubation. [file 40104_2021_614_MOESM1_ESM.docx]

Supplemental Table 1. Relative abundance of predicted KEGG level 2 (Top 20) affected by endolysin LyJH307 in an *in vitro* experiment at 12 h of incubation

|  | Treatments^1)^ | | |  |  |
| --- | --- | --- | --- | --- | --- |
| Items^*^ | CON | | LyJH307 | SEM | *P*-value |
| Metabolism | | 14.327 | 14.172 | 0.0774 | 0.3827 |
| Carbohydrate metabolism | | 14.327 | 14.172 | 0.0774 | 0.3827 |
| Metabolism of cofactors and vitamins | | 14.175 | 14.142 | 0.0904 | 1.0000 |
| Amino acid metabolism | | 12.936 | 12.774 | 0.1043 | 0.1904 |
| Metabolism of terpenoids and polyketides | | 8.730 | 8.553 | 0.1891 | 1.0000 |
| Metabolism of other amino acids | | 6.989 | 7.299 | 0.3180 | 0.6625 |
| Glycan biosynthesis and metabolism | | 5.675 | 5.563 | 0.0705 | 0.1904 |
| Energy metabolism | | 5.535 | 5.484 | 0.2104 | 1.0000 |
| Lipid metabolism | | 4.439 | 4.416 | 0.0622 | 0.6625 |
| Biosynthesis of other secondary metabolites | | 2.684 | 2.626 | 0.0099 | 0.0809 |
| Xenobiotics biodegradation and metabolism | | 1.885 | 2.193 | 0.3972 | 1.0000 |
| Nucleotide metabolism | | 2.197 | 2.166 | 0.0177 | 0.0809 |
| Genetic information processing - | |  |  |  |  |
| Replication and repair | | 6.348 | 6.281 | 0.0729 | 1.0000 |
| Translation | | 3.517 | 3.481 | 0.0404 | 0.6625 |
| Folding, sorting and degradation | | 3.050 | 3.076 | 0.0238 | 0.3827 |
| Transcription | | 1.193 | 1.144 | 0.0280 | 0.1904 |
| Cellular processes | |  |  |  |  |
| Cell growth and death | | 1.737 | 1.728 | 0.0087 | 0.6625 |
| Cell motility | | 1.616 | 1.751 | 0.0771 | 0.3827 |
| Transport and catabolism | | 0.269 | 0.262 | 0.0051 | 0.1904 |
| Environmental Information processing | |  |  |  |  |
| Membrane transport | | 1.473 | 1.541 | 0.0420 | 0.3827 |
| Signal transduction | | 0.262 | 0.272 | 0.0057 | 0.3827 |

* SEM, standard error of the mean; KEGG, Kyoto Encyclopedia of Genes and Genomes; COG, clusters of orthologous genes; EC, enzyme classification.

^1)^ CON, corn grain with elution buffer of the same volume used endolysin treatment; LyJH307, corn grain with recombinant LyJH307 (0.2% of dietary DM).
